# PCDH19-Related Epilepsy in Early Onset of Chinese Male Patient: Case Report and Literature Review

**DOI:** 10.3389/fneur.2020.00311

**Published:** 2020-04-30

**Authors:** Xiao Yang, Jing Chen, BiXia Zheng, Xianyu Liu, Zixuan Cao, Xiaoyu Wang

**Affiliations:** Department of Neurology, Children's Hospital of Nanjing Medical University, Nanjing, China

**Keywords:** PCDH19, gene mutation, epilepsy, male, neurology

## Abstract

Mutations in *PCDH19* are associated with epilepsy, intellectual disability and behavioral disturbances, mostly related to females. The unique X-linked pattern of inheritance affects females predominantly, while usually is transmitted through asymptomatic males. Recently, new research has demonstrated that males with a mosaic pattern of inheritance could also be affected. As yet, *PCDH19* mutations have been reported in hundreds of females; however, only 15 mosaic males were reported to exhibit epileptic seizures with the onset ranges between 6 and 31 months. These patients were usually reported to carry various mutations in the *PCDH19*. Here we describe a non-sense variant at the *PCDH19* (c.498C>G; p.Y166^*^) in the Chinese male that exhibited early developmental delay and frequent seizures starting from the age of 5 months. We aim that this case report, focusing on studying clinical seizures, therapeutic approaches, and the patient's prognosis, will contribute to the cumulative knowledge of this rare and complex genetic disorder.

## Introduction

In 1971, Juberg reported a unique inherited epilepsy in a family from North American, in which 15 females seizured at different ages (range from 6 to 18 months) and no male patient was diagnosed (1). Later in 1997, Ryan proposed a X-linked theory to be responsible for the female-related heredity (2). Then the *PCDH19* mutation was first reported to be associated with epilepsy and intellectual disability limited to the females (EFMR) in a family in 2008 and proposed as the cause of epilepsy by Dibbens et al. (3).

The gene is located on the long arm of the X-chromosome (Xq22.1) and encodes for protocadherin-19. The location of the gene assumes an unusual X-linked female exclusive inheritance (4). Most of the known mutations are located in the first exon that encodes the extracellular domain of the protein (5, 6). In 2012, Depienne and LeGuern reported the synergetic effect the protocadherin 19 may have with N-cadherin (NCAD) during anterior neurulation in zebrafish. This study found that the protocadherin 19 forms a *cis*-complex with NCAD, which was shown to play an important role in the regulation of cell motility during brain development. Within the complex, protocadherin 19 plays a dominant part, while NCAD is mostly acting as an essential *cis*-cofactor (5). It has been proposed that the mutations in *PCDH19* has resulted in the loss of the protein function lead to the disruption of the cell-cell adhesion, which could be the underlying reason for the seizures (7). Other than the cellular interference hypothesis, Higurashi proposed the dysfunction of blood-brain barrier (BBB) could be another cause of *PCDH19*-related epilepsy (8). They found seizures resulting from *PCDH19* mutations commonly originate in the limbic region, where it is more closer to the periventricular regions without the BBB. Furthermore, deficiency of some hormones or metabolites caused by these abnormal genes is considered to induce seizures (9). Decrease of allopregnanolone and neuroactive steroids in female patients with *PCDH19*-related epilepsy can provide strong evidence and further studies of other neurosteroids are needed (10).

Until recently, Cellular Interference was considered to have been associated with the inheritance pattern in which heterozygous females are affected while hemizygous males are healthy (11). However, recent findings show that mosaic male carriers of the mutated *PCDH19* may also be affected (12–14). To date, more than 160 mutation sites on *PCDH19* in nearly 400 female patients have been detected whereas male patients are significantly rare (11, 12). In this article, we report one male patient with a non-sense variant of the *PCDH19* (c.498C>G; p.Y166^*^) and summarized other reported male cases for further study on clinical seizures, onset ages, drug treatment and disease prognosis.

## Case Report

A two-and-half-year-old male was reported to suffer from repetitive seizures starting the age of 5 months. He was born at full-term via normal vaginal delivery without birth-related distress or dysmorphia. Familial and personal neurologic antecedents were negative. Perinatal history and pregnancy were both unremarkable. Except for mildly laryngeal cartilage dysplasia, no other serious congenital diseases of the neonatal period were reported prior to the appearance of the first seizure at the age of 5 months. The first seizure appeared as a tonic seizure following by screaming and a frightened face, including loss of contact, stiff limbs, purple lips, and chewing movements. These onsets usually started during sleep and continued for about 20–30 s with or without a high fever. A cluster of focal seizures appeared with slight fever after 4 months' seizure-free and frequent attacks, this made the patients seek help from the hospital. EEG showed sharp waves, and an MRI scan suggested a widened bilateral frontotemporal gap. Valproate (VPA) treatment started, then tonic seizures still existed from time to time. Levetiracetam (LEV) was added in his treatment after 6 month of continuous seizures, and later of topiramate (TPM). TPM was later replaced by phenobarbital (PB) due to the serious side effect of hypohidrosis. The patient experienced reduced attack frequency after the regimen change; however, the damage to the liver functions forced the withdrawal of VPA starting from August 2019. On August 30, 2019, the patient had another cluster of seizures, which led to the hospitalization and an addition to the regimen of dexamethasone (DXMS) and clonazepam (CZP). The latest reported onset occurred in September 2019. The appearance of epilepsy seizures accompanied by a distinct developmental delay and intellectual disability(ID). No scales had been used for accurately assessing patient's intelligence level and mental disorder whether before, or after his onset. But during the follow-up period, his parents found increased improvement in language communication and physical activity.

**Figure 1:**
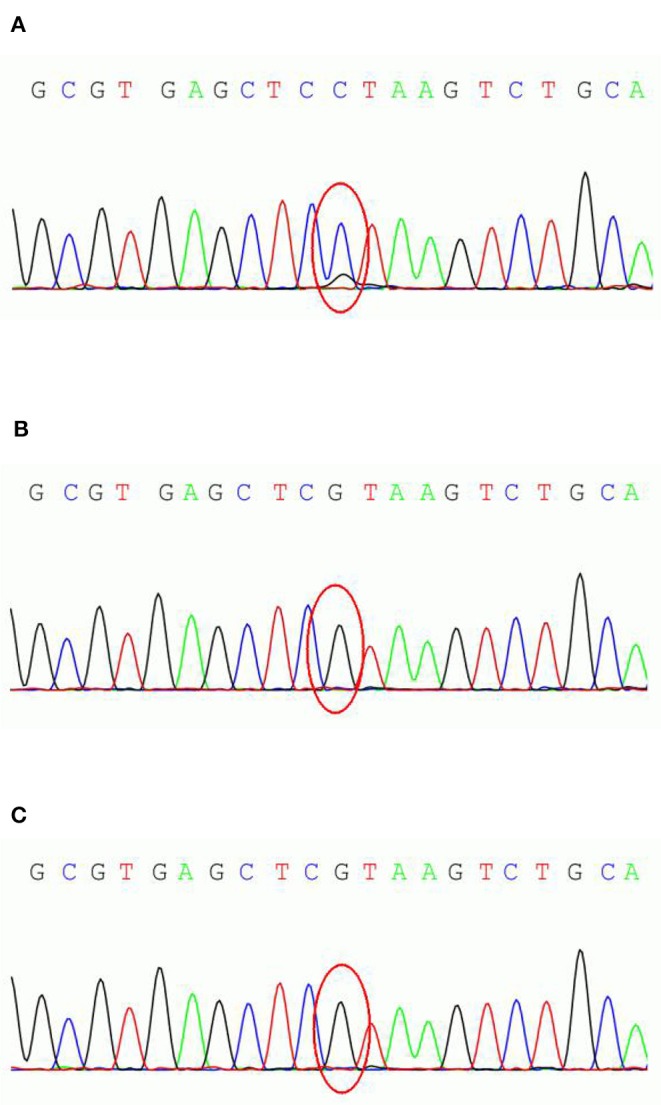
*PCDH19* sequencing results of the parent-proband trio. **(A)** the male patient; **(B)** patient's father; **(C)** patient's mother.

We conducted genetic testing (exon sequence) of the patient and his parent's DNA, extracted from the peripheral blood leukocytes, with the cooperation of Running Gene, Beijing, China. Gene analysis revealed a suspected mosaic mutation (c.498C>G) in the exon region (NM_001184880, exon1) of the *PCDH19* of the patient but not of his parents (Figures 1A–C). This non-sense mutation results in the early termination of protein translation and significantly affects protein function. According to the guide of The American College of Medical Genetics and Genomics (ACMG), this gene mutation could receive a pathogenic rating.

## Review of Male Patients With PCDH19 Mutations

We used “PCDH19” and “male” as key words to accomplish our literature retrieval in Pubmed. Further reviewing of the literature has detected 14 male *PCDH19*-related epilepsy cases from eight reports that were integrated with the current case for comparison analysis of the parameters that included the onset age, clinical seizures, variant positions, and treatment. The purpose of this study was to investigate whether *PCDH19*-related epilepsy has characteristic similarities among the affected males. The study results are summarized in Table 1 (11, 15–18).

**Table 1:** Clinical manifestation of 15 male patients with PCDH19 gene variants.

**Proband no**.	**Sex**	**Sz age (months)**	**Sz types**	**Fever sensitivity**	**Seizure clusters**	**Effective AEDs**	**Age at last follow-up(year)**	**Development prior to onset**	**Intellectual disability**	**Motor delay**	**Behavior al problems**	**Gene variants**
1	M	12	FS, GTCS, prolonged, repetitive	+	+	VPA, CLB, CZP, TPM, Stiripentol	7	normal	+	+	Autistic features	del PCDH19
2	M	9	FS, tonic-clonic, FSsG	?	?	LEV, ZNS, et al.	6	normal	+	+	Irritability, aggression, rigidity, poor sleep, ADHD, anxiety, OCD	c.605C>A p.Ser202*
3	M	9	FS, focal tonic-vibratory	+	+	VPA	4	normal	+	?	Compulsive and stereotyped behavior	c.918C>G p.Tyr306*
4	M	10	Fs, tonic, often fearful screaming at start	+	+	PHB, VPA	3, 5	normal	-	-	-	c.1352C>T p.Pro451Leu
5	M	11	Myoclonic, tonic-clonic	?	+	PB, LEV	8	normal	+	+	ADHD, autism	c.2147+2 T>C
6	M	5	CP, secondary generaliaed. Focal with affective symptoms, secondary generalized	+	+	LEV, OXC, CLB, TPM	10	normal	Languages delay	+	Autism, aggression, ADHD	c.1864G>C p.Gly622Arg
7	M	10	Generalized clonic	+	+	VPA	13	normal	-	-	Autism, mood disorder	c.840C>G p.Tyr280*
8	M	10	Cluster of CP seizures, secondary tonic clonic, status	+	+	LEV	14	normal	+	+(balance problems)	Autism spectrum disorder, anxiety	c.462C>G p.Tyr154*
9	M	7	Focal, febrile, GTC, tonic, CP	+	+	OXC, VPA, PHB, LEV	2	normal	Speech development delay	+(balance problems)	?	c.1682C>G p.Pro561Arg
10	M	31	CP, febrile	+	+	OXC	13	Speech delay	Midly delayed speech development	-	Behavior problems resembling autism spectrum disorder, short attention span	c.799G>T p.Glu267*
11	M	16	Focal and geneliazed seizures	+	+	?	5, 5	Speech delay from 12 months	Speech delay	-	?	c.706C>T p.Pro236Ser
12	M	7	Generalized tonic seizures,	+	+	VPA, PB, OXC	2, 3	normal	+	+	?	c.1508-1509inst p.Thr504HisfsTer19
13	M	9	GTCs, absence seizure, myoclonic seizures	+	+	VPA, TPM	9	normal	+	?	Aggression, autism spectrum disorder	c.158dupT p.D54GfsX35
14	M	5	Focal	-	+	OXC, LEV, VPA, TPM	3	normal	+	?	Agression	c.317T>A p.M106R
15	M	5	Fs, tonic, often fearful screaming at start	-	+	LEV, PB, CZP	2, 6	normal	+	+	?	c.498C>G chrX-99663098 p.Y166*

*M, male; FS, focal seizure; GTCS, generalized tonic-clonic seizure; FSsG, focal seizure with secondary generalization; CP, partial complex;AED, anti-epileptic drug;VPA, valproate; CBZ, carbamazepine; CLB, clobazam; LEV, levetiracetam; LTG, lamotrigine; OXC, oxcarbazepine; TPM, topiramate; ADHD, attention deficit hyperactivity disorders*.

All patients included in this table were males with median onset age of 9.5 months (range 5–31 months). *PCDH19* mutation positions contain c.158dupT(p.D54GfsX35), c.317T>A(p.M106R), c.462C>G (p.Tyr154^*^), c.498C>G(p.Y166^*^), c.605C>A(p.Ser202^*^), c.799G>T(p.Glu267^*^), c.706C>T(p.Pro236Ser), c.840C>G (p.Tyr280^*^), c.918C>G (p.Tyr306^*^), c.1352C>T (p.Pro451Leu), c.1508-1509insT(p.Thr504HisfsTer19), c.1682C>G (p.Pro561Arg), c.1864G>C(p.Gly622Arg), c.2147+2T>C. Case 15 refers to the current study at which the patient possesses the same mutation position that was reported in a female patient by Smith L (16). Smith showed that the seizures onset age of the female patient was 10 months, while in our case 15, the disease onset had occurred significantly earlier (5 months). Repeated and uncontrolled seizures of the female patient still existed while variety of antiepileptic drug combination therapy had been used. Long-term follow-up found clear development disability and sleep disorders which had become a serious situation. In the female patient, a focal seizure was the only seizure type, while the male case included complex seizure types such as tonic, tonic-clonic, myoclonic, and generalized tonic-clonic seizures (GTCS). Nearly all of the cases showed more than one seizure type during the disease progression, while only one patient has been free of seizures (case 13). In both cases 4 and 15, a unique fearful screaming was present at the beginning of seizures. Fever sensitivity and seizure clusters were prevalent in 73% of male cases (11 of 15). Following the latest evaluation, in several cases, the patients were treated with more than three medicaments; however, the management of symptoms remained poor-controlled.

In one patient (case 8), treatment with levetiracetam (LEV) was reported as profoundly effective. In other patients, treatment with oral polypharmacy, clonazepam (CZP) and phenobarbital (PB) also was reported as effective. Most cases (13 of 15) were reported as having normal development before seizures' onset, however almost all of the patients exhibited ID or motor delay (delayed expression and balance disorder) in the later stage (except cases 4 and 7). Many patients reported to have a broad spectrum of behavior disturbances in high incidence. These include autism (7 of 15), aggression (4 of 15), and attention deficit hyperactivity disorder (ADHD) (2 of 15).

## Discussion

Molecular genetic screening has become an effective path for defining diagnosis or searching for the cause of some epilepsy and epilepsy syndromes, especially in the identification of epilepsy syndromes with similar clinical manifestation. In previous reports, some fever-related epilepsy patients were found *SCN1A*-negative and were diagnosed as DS-like. Targeted gene detection on *PCDH19* should be reconfirmed in patients with the following features: age at onset before 3 years, seizures with earlier fearful screaming, cluster occurrence and cognitive and behavior disturbances (19). These hallmarks of *PCDH19*-related epilepsy both happened in female and male patients and there is no characteristic differences between two genders. The patient present here has a reported mutation site while his onset age was much earlier than the female case. For male patients, mosaic condition should be considered as possible genetic cause and further functional analyses are needed to prove the pathogenicity in male cases. Depienne used according to the suggested use, could possibly lower the percentage of mosaicism in male patients and this has led to a milder phenotype (5). This assumption cannot be confirmed due to the limited clinical evidence.

Disorders on cognitive development or psychiatric symptoms has become a common situation among patients with *PCDH19*-related epilepsy. Rarely cases show developmental delay before seizure onsets. Their impairment usually appears after the age of two and is not known to have a connection with seizure severity. Our case had no developmental issues before seizure onset and therefore the typical cognitive delay and motor disability could be observed after seizure onset. Although original antiepileptic drug-treatment was not working well, his developmental delay still improved progressively over time. This feature could also be noticed in other typical developing patients (20). Detailed cognitive assessment should be used to estimate cognitive level of patients, such as Griffiths Mental Developmental Scales and Wechsler scales. Autism is the most common psychiatric symptom accompanied with *PCDH19*-related epilepsy, accounted for ~20% of patients described by a systematic review (21). The other symptoms include anxiety, obsessive-compulsive disorders, and oppositional defiant disorders. One-fifth of them were defined with multiple psychiatric comorbidities. High incidence of cognitive delay and psychiatric symptoms guided crux of early psychological assessment and followed behavioral therapy.

Just as our summary showed above, most *PCDH19*-related epilepsy patients needed polytherapy with various combinations and none have proved definitively superior. Several patients with brain lesions had seizure control improved after surgery while most of all is refractory to antiepileptic treatment (22). Jan et al. reported a retrospective multicenter study of antiepileptic therapy in 58 female patients with *PCDH19* mutations (11). After 3 months of treatment, bromide and clobazam were affirmed the most effective drugs with a responder rate of 68% and 67%. After 1-year of treatment, bromide and clobazam, respectively, with the responder rate of 50 and 43%. Three quarters of the patients became seizure-free for at least 3 months, and half of them for at least 1 year. At the same time, positive effective of sodium channel blockers would help us to understand the mechanism of epileptogenesis in PCDH19 mutations (23). Further studies should put more attention on precision therapies which can target underlying pathogenesis, in order to improve epilepsy seizure as well as neuropsychiatric symptoms in these patients.

## Conclusion

Here we reported a Chinese male patient with a non-sense variant on PCDH19(c.498C>G; p.Y166^*^). He had a much earlier onset age with typical clinical features and obvious cognitive developmental disorders, which would usually be seen in female patients reported by other scholars. Early onset age of epilepsy seizure might confuse clinical doctors to a wrong diagnosis. While we discover typical features about PCDH19-related epilepsy, molecular genetic screening can further clarify possible epilepsy syndrome. Early psychological assessment along with antiepileptic drugs treatment and followed by behavioral therapy would be essential for recovering social functions. Several adjustments of oral drugs in our case reminded awareness of side effects. Although epilepsy in *PCDH19* mutations is often cognitive pharmacoresistant, bromide and clobazam are expected to be effective in long-term treatment of antiepileptic drugs. Until the latest follow-up, combination therapy of DXMS and clonazepam had effect seizure control and longer observation is needed to estimate long-term effectiveness and potential side effects.

## Data Availability Statement

The raw data supporting the conclusions of this article will be made available by the authors, without undue reservation, to any qualified researcher.

## Ethics Statement

Written informed consent was obtained from the individual(s), and minor(s)' legal guardian/next of kin, for the publication of any potentially identifiable images or data included in this article.

## Author Contributions

XY wrote the article with guidance of JC, while BZ perfected the contents of genetics. XL, ZC, and XW had collected relevant case information.

## Conflict of Interest

The authors declare that the research was conducted in the absence of any commercial or financial relationships that could be construed as a potential conflict of interest.
